# Microaneurysm segmentation in early diabetic retinopathy: a localized patch-based U-Net approach to overcome microscopic lesion oversight

**DOI:** 10.1186/s40942-026-00919-x

**Published:** 2026-08-14

**Authors:** Likhitha D. Atada, Madhura Prakash Manjunath, Deepthi K. Prasad, Venkatakrishnan Srinivasan

**Affiliations:** Forus Health Pvt Ltd., 8, 27th Cross Rd, Banashankari Stage II, Banashankari, Bengaluru, Karnataka 560070 India

**Keywords:** Diabetic retinopathy, Early DR, Fundus imaging, Medical image analysis, Microaneurysm segmentation, Patch-based deep learning, U-Net

## Abstract

**Background:**

Diabetic Retinopathy (DR) is a leading cause of vision impairment and a major long-term microvascular complication of diabetes. Early detection and prevention of diabetes-related complications through advanced imaging and software-assisted patient management remain important clinical priorities. Microaneurysms (MAs) are the earliest and most subtle indicators of DR, but their small size and low contrast often lead to missed detection during manual fundus examination, delaying intervention. Automated MA segmentation is therefore essential for large-scale DR screening.

**Methods:**

We conducted a controlled empirical evaluation of localized patch-based training for microaneurysm (MA) segmentation, benchmarking a compact, imbalance-aware U-Net model including learnable transposed-convolution up-sampling against a full-image U-Net trained on identical data. Fundus images and corresponding MA masks were divided into non-overlapping 256 × 256 patches, increasing MA pixel density per training sample by approximately 60-fold relative to full-image input thereby directly targeting the extreme class imbalance that causes full-image models to collapse to all-background predictions. The model was trained and evaluated on the publicly available IDRiD [Indian Diabetic Retinopathy Image Dataset] and DDR [Dataset for Diabetic Retinopathy] datasets. Performance was assessed using Intersection over Union (IoU), Dice coefficient, accuracy, recall, and precision.

**Results:**

Performance was evaluated on a representative held-out subset of 100 images selected from an independent pool of 514 MA-annotated test images spanning the IDRiD and DDR datasets. The patch-based model achieved an overall pixel-level accuracy of 99.89%, a Dice coefficient of 76.9%, IoU of 63.2%, recall (sensitivity) of 69.1% and precision of 88.7%, while the full-image U-Net trained on the same data failed to recover any MA pixels (IoU = 0, Dice = 0) despite comparable pixel accuracy thereby demonstrating that overlap-based metrics, not accuracy, are the decisive criterion for this task. Per-image lesion-coverage analysis showed a mean match rate of approximately 60% of annotated MA contours, providing a clinically interpretable read-out beyond pixel overlap.

**Conclusion:**

This controlled empirical evaluation demonstrates that localized patch-based training is an effective and computationally efficient strategy for overcoming the extreme class imbalance that causes conventional full-image U-Nets to systematically miss microaneurysms. The proposed patch-based U-Net showed promising microaneurysm segmentation performance on a held-out subset of IDRiD and DDR images; further evaluation on the additional datasets and independent external cohorts can aid in the clinical screening utility.

## Introduction

Diabetic Retinopathy (DR) is a leading cause of preventable blindness worldwide. It begins with subtle changes in the retinal microvasculature and progresses through the formation of microaneurysms (MAs), hemorrhages, and exudates. A recent Global Burden of Disease analysis estimated that the number of people blind due to DR increased by 326.0% between 1990 and 2021 [1]. Because MAs are the earliest and most specific markers of early DR, their accurate detection is pivotal for timely diagnosis and therapeutic intervention. In routine fundus examination, however, MAs are frequently overlooked due to their diminutive size (typically < 125 μm in diameter), their low contrast against the surrounding retinal background, and the inherent subjectivity and inter-rater variability of manual grading. Automated, objective, and reproducible detection methods are therefore increasingly important for population-level DR screening.

Deep learning, and in particular Convolutional Neural Networks (CNNs), has transformed medical image analysis and demonstrated strong performance on multiple retinal imaging tasks. Nonetheless, accurately segmenting extremely small lesions such as MAs remains challenging. Standard pixel-wise and full-image segmentation models often struggle with these fine structures because of:

- **Low lesion contrast and high inter-class similarity —** MAs share color statistics with neighboring retinal tissue, predisposing pixel classifiers to mis-label them as background.
- **Loss of small-scale features —** successive down-sampling stages in deep CNNs can erase sub-pixel structures before they are ever decoded.
- **Class imbalance —** MA pixels represent a vanishingly small fraction of the total fundus image, so a naively trained model can attain high accuracy while ignoring lesions entirely.

### Related work

#### From feature engineering to hybrid models

Early MA-detection pipelines relied on hand-crafted features coupled with classical machine-learning classifiers. Multi-scale Hessian-based blob detectors [2], gradient-vector analysis [3] and image transforms such as the Pixel Intensity Rank Transform (PIRT) [4] have all been used to isolate candidate MAs, which were then classified using models such as Naive Bayes with features like Directional Local Contrast (DLC) [5]. Hybrid approaches further integrated fuzzy logic with Particle Swarm Optimization to delineate ambiguous regions [6], while specialized boosting classifiers such as Random Under-Sampling Boost (RUSBoost) were introduced to mitigate the extreme class imbalance characteristic of MA datasets [3].

#### Deep learning for MA detection

Contemporary research is dominated by CNN-based models. Patch-based classification approaches train a CNN on small image windows to discriminate MA-bearing regions [7, 8]; hybrid models embed features from multiple pre-trained backbones, e.g. VGG-19 and Inception-v3, for improved robustness [9]; and sliding-window pipelines apply CNNs trained on a small number of annotated patches to generate lesion probability maps across entire fundus images [10]. Encoder–decoder architectures — most prominently U-Net and its variants — have become the de-facto standard for lesion segmentation, with lightweight Attention U-Nets reducing parameter count while improving performance [11]. Complementary innovations include attention mechanisms that emphasize salient features in shallow layers [12], Dilated Residual Networks that preserve fine spatial detail [13] and object-detection frameworks adapted for MA detection such as MA-YOLO, which combines super-resolution (SwinIR) with Wise-IoU loss for improved small-object localization [14].

#### Core challenges and clinical context

Across methodologies, preprocessing remains pivotal: green-channel extraction and contrast enhancement techniques such as CLAHE are near-universal steps to improve MA visibility [2, 11]. Class imbalance is tackled via tailored training strategies including two-step CNNs that pre-select difficult samples [15] and reinforcement-style retraining on high-error batches [8]. Beyond pure detection, Dubow et al. used high-resolution AOSLO (Adaptive Optics Scanning Light Ophthalmoscope] fluorescein angiography to establish a six-type morphological MA classification and linked morphology to physical size, providing a clinically informed framework for risk assessment [16]. Together, these studies highlight a progressive shift from detection-only algorithms toward clinically interpretable, context-aware tools.

#### Patch-based U-Net precedents

Patch-based training combined with U-Net-family segmentation has previously been applied to MA detection. Raudonis et al. proposed a three-stage pipeline — image decomposition into patches, inference by an ensemble of U-Net, ResNet34-UNet, and UNet++, and reconstruction of the full-resolution segmentation map — reporting Dice 0.95 and IoU 0.91 [17]. Platas-Campero et al. applied patch decomposition with a statistical homogeneity filtering step ahead of U-Net training and evaluated inter-disease out-of-domain generalization from diabetic to leukemic retinopathy, reporting Dice 0.914 and IoU 0.842 on IDRiD [18]. Attention-augmented U-Net variants have also been reported for this task: Vanaja and Prakasam’s CBAM-AG-UNet achieved Dice 0.865 and IoU 0.758 on IDRiD [19], while Monisha Birlin et al.‘s frequency-spatial-channel attention U-Net (FSCA-UNet) reported Dice of 0.4481, 0.4860, and 0.3561 on IDRiD, E-Ophtha, and DDR respectively [20], illustrating substantial performance variability across MA-segmentation studies depending on dataset, split, and preprocessing. The present study differs from these in evaluating patch-based training against a matched full-image baseline on the identical cohort, rather than reporting patch-based results in isolation.

Recent advances in microaneurysm (MA) detection continue to refine U-Net architectures, incorporate transformer-based global context modeling, and expand multi-modal imaging applications. Bida and Naser investigated automated MA detection using a Salp Swarm Algorithm (SSA)-optimized U-Net coupled with a Bayesian-optimized CNN [21], while Tahir and Zhang introduced a lightweight Attention U-Net incorporating an optimized preprocessing pipeline to handle low-contrast retinal fundus images [22]. In parallel, Kou et al. developed a deep recurrent U-Net (DRU-Net) that integrates deep residual learning and recurrent convolutional operations to enhance feature accumulation for small MA structures [23]. Addressing multi-modal alternatives, Husvogt et al. implemented U-Net ensembling strategies for MA segmentation using optical coherence tomography angiography (OCTA) en face projections from both superficial and deep capillary plexuses [24]. Furthermore, broader segmentation paradigms such as Santone et al.’s U-Net deployment for general retinal feature extraction [25] and Vanaja and Prakasam’s CBAM-attention gated U-Net frameworks [26] underscore the ongoing focus on attention mechanisms and encoder-decoder refinements for clinical screening.

The comprehensive summary of literature is provided in Table 1.

**Table 1:** Summary of related-work approaches for microaneurysm detection and segmentation

Approach / Reference	Key Advantage	Disadvantage / Limitation	Summary
Particle Swarm Optimization (PSO) [6]	Effectively optimizes parameters for fuzzy logic rules to delineate ambiguous retinal regions.	High computational overhead during swarm convergence.	High boundary accuracy on ambiguous lesions.
Multi-Scale Hessian Blob Detectors [2]	Isolates candidate structures across multiple scales without exhaustive window scanning.	Sensitive to background noise and false positives from vessel intersections.	High candidate recall rate.
Directional Local Contrast (DLC) + Naive Bayes [5]	Low complexity feature extraction tailored for directional contrast changes.	Relies heavily on hand-crafted thresholds which fail under uneven illumination.	Efficient classification speed for binary candidate splits.
Pixel Intensity Rank Transform (PIRT) [4]	Robust against minor background lighting variations and intensity shifts.	Loses absolute intensity nuances critical for subtle lesions.	Reliable candidate isolation under contrast shifts.
Patch-Based CNN Windows [7, 8, 10]	Directly learn localized lesion features from small image patches.	Discards global anatomical context; prone to high false-positive rates on normal background patches.	High patch-level discrimination AUC.
Two-Step / Reinforcement CNNs [8, 15]	Explicitly targets hard-to-classify samples and high-error batches via iterative retraining.	Training pipeline is complex and computationally expensive.	Improved precision on challenging low-contrast datasets.
Dilated Residual Networks [13]	Expands receptive fields while preserving fine spatial detail without pooling loss.	Requires carefully tuned dilation rates to avoid gridding artifacts.	Enhanced spatial resolution retention.
Hybrid Backbone Ensembles (VGG-19 & Inception-v3) [9]	Combines multi-scale feature hierarchies from diverse pre-trained networks.	Excessive model size and heavy memory footprint.	Robust cross-dataset feature generalization.
SwinIR + YOLOv8 (MA-YOLO) [14]	Super-resolution integration with specialized loss (Wise-IoU) for tiny object localization.	High inference latency due to super-resolution preprocessing.	Superior small-object localization accuracy.
Attention-Mechanism CNNs [12]	Emphasizes salient spatial features explicitly within shallow layers.	Adds parameter overhead to basic convolutional blocks.	Enhanced focus on faint lesion features.
Gradient-Vector Analysis + RUSBoost [3]	Explicitly addresses extreme class imbalance characteristics of microaneurysm datasets.	Boosting ensemble training can be slow on large high-resolution datasets.	Balanced sensitivity and specificity on imbalanced data.
AOSLO Fluorescein Angiography Morphology [16]	Establishes a clinical 6-type morphological classification linked to physical size.	Invasive imaging modality requiring specialized, expensive clinical equipment.	High clinical correlation for risk assessment.
Lightweight Attention U-Net [11, 22]	Reduces parameter count while maintaining high segmentation accuracy on low-contrast scans.	May lose fine details if downsampling is overly aggressive.	High inference efficiency and early diagnosis metrics.
CBAM-Attention Gated U-Net [19, 26]	Suppresses irrelevant background features and highlights fine lesion boundaries.	Sensitive to heavy noise and uneven illumination in unenhanced images.	Dice: 0.865, IoU: 0.758 (IDRiD)
Statistical Homogeneity + U-Net [18]	Enables out-of-domain generalization across different retinopathies (diabetic to leukemic).	Pre-filtering steps can occasionally discard low-contrast true positives.	Dice: 0.914, IoU: 0.842 (IDRiD)
Frequency-Spatial-Channel Attention U-Net (FSCA-Net) [20]	Multi-domain feature extraction using frequency and spatial channel attention.	Performance fluctuates widely across multi-center datasets.	Dice: 0.448 (IDRiD), 0.486 (E-Ophtha), 0.356 (DDR)
Ensemble U-Net / ResNet34-UNet / UNet**++** [17]	Multi-architecture consensus reduces individual model bias and segmentation errors.	Extremely demanding computational requirements for multi-model inference.	Dice: 0.95, IoU: 0.91
SSA-Optimized U-Net & Bayesian CNN [21]	Enhanced hyperparameter optimization and feature exploration via metaheuristics.	High computational complexity during the optimization phase.	Robust convergence across metrics.
Deep Recurrent U-Net (DRU-Net) [23]	Integrates deep residual learning and recurrent operations for feature accumulation.	Increased memory footprint due to recurrent operational layers.	Strong small-object sensitivity on benchmark fundus datasets.
Standard U-Net Retinal Extraction [25]	Simple, modular backbone adaptable to generic vascular and lesion segmentation tasks.	Lacks specialized self-attention mechanisms for extremely small scale anomalies.	Baseline vascular/lesion layout extraction accuracy.
Ensembled OCTA U-Nets [24]	Leverages volumetric and layered blood flow data (superficial and deep capillary plexuses).	Dependent on high-resolution OCTA equipment availability.	High volumetric segmentation reliability.

### Contributions of this work

Despite these advances, the combined challenge of tiny lesion size, low contrast and extreme class imbalance continues to challenge the reliability of automated MA segmentation. In this work, we present a controlled empirical evaluation of a customized U-Net segmentation model trained under a patch-based strategy that explicitly addresses these limitations. This work utilized established components of the segmentation literature such as Batch Normalization, LeakyReLU, transposed convolution, and skip connections; the contribution is the quantified, matched-cohort demonstration of their combined effect for MA segmentation, detailed below. Unlike prior CNN-based MA detectors that either operate on whole-image inputs or perform patch-level classification, our work combines a fully patch-based semantic-segmentation strategy with a streamlined, imbalance-aware U-Net (BatchNorm + LeakyReLU) and loss‐function, yielding superior small-lesion delineation and computational efficiency under extreme class imbalance. The contributions in detail are mentioned below:

1. **Localized patch-based semantic segmentation.** Fundus images and their paired MA masks are systematically decomposed into non-overlapping 256 × 256 patches. By training on patches rather than full images, the network focuses on localized regions, refines lesion boundaries, improves discriminability of small low-contrast structures and mitigates class imbalance.
2. **Customized U-Net architecture.** The U-Net is adapted for MA detection through Batch Normalization and LeakyReLU activations for stable training, skip connections to preserve spatial information, transposed convolutions for learnable up-sampling and a reduced filter budget (32→64→128→256) that reduces computational cost without sacrificing accuracy.

#### Training and evaluation protocols

Dice Loss and Focal Loss were assessed alongside MSE during development; MSE was retained based on preliminary observations of training stability, and this choice is revisited as an open question in section “Training strategy and optimization” and section “Discussion” rather than presented as a settled comparison. We assessed performance using clinically relevant metrics such as IoU, Dice coefficient, pixel accuracy, recall, and precision. Results were benchmarked against a standard full-image U-Net; section “Ablation scope and analytical justification of component contributions” details the scope and limits of this comparison.

We note explicitly that localized patch-based training and a systematic training/evaluation protocol are established practices in medical image segmentation generally and have previously been applied to MA segmentation specifically (see Related Work). This study’s contribution is the quantified, matched-cohort demonstration of their effect for MA segmentation, together with an analytical accounting of what can and cannot be attributed to each design choice (section “Ablation scope and analytical justification of component contributions”).

Together, the proposed patch-based strategy, customized U-Net and rigorous evaluation yield a more reliable foundation for early-DR screening. The remainder of this paper is organized as follows. Section “Materials and methods” describes the datasets, preprocessing pipeline, model architecture, training protocol, and evaluation of metrics. Section “Results” reports quantitative and qualitative results and compares the proposed model against a customized U-Net. Section “Discussion” discusses clinical implications and limitations, and section “Conclusion and future work” concludes the paper and outlines directions for future work.

## Materials and methods

### Datasets

The proposed framework was developed and evaluated on two publicly available fundus datasets with pixel-wise MA annotations: the Indian Diabetic Retinopathy Image Dataset (IDRiD) [27] and the Dataset for Diabetic Retinopathy (DDR) [28].

**Fig. 1:**
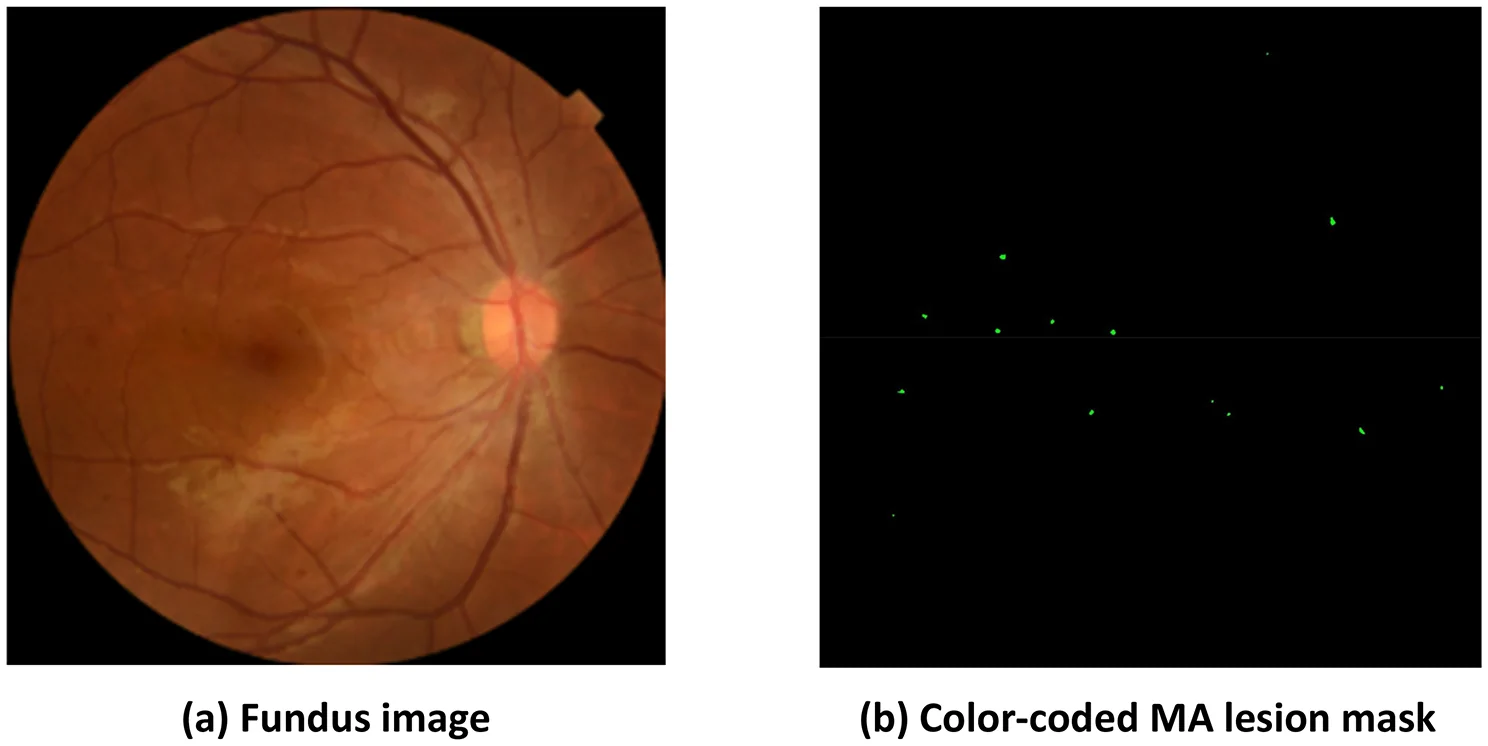
Fundus image (left) and corresponding MA lesion mask rendered in green [RGB (0, 255, 0)] (right)

For visualization and training consistency, a standardized color encoding of pure green [RGB: (0, 255, 0)] was assigned to MA lesions in the segmentation masks. A representative fundus image and its color-coded MA lesion mask are shown in Fig. 1.

While the combined IDRiD and DDR dataset comprise 838 annotated images, experiments were restricted to a curated subset of 215 images (115 for training, 100 for testing) due to severe hardware bottlenecks encountered during dense spatial training. We confirm that the train/test split was performed at the image level prior to patch extraction, so that all patches derived from a given source image belong exclusively to either the training set or the test set; this precludes patch-level leakage across the split. All pipelines were executed on an Azure Standard_NC6s_v3 virtual machine equipped with a single NVIDIA Tesla V100 GPU (16 GB VRAM).

The decision to limit the immediate cohort size was driven by the memory footprint generated by high-resolution retinal images combined with dense patch extraction and full-resolution feature mapping. Specifically, fundus images captured at clinical resolutions (ranging from 1024 × 1024 up to 2048 × 2048 pixels) scale exponentially in memory during tensor backpropagation. Partitioning each image into non-overlapping 256 × 256 patches yields an immense data cardinality—where a single 2048 × 2048 image produces 64 distinct spatial patches, resulting in an active training pool of approximately 8,000 high-resolution sub-regions from just 100 images. Attempting to scale this pipeline to the full 838-image corpus triggered persistent CUDA out-of-memory (RESOURCE_EXHAUSTED) errors on the 16 GB VRAM ceiling, even with optimized gradient accumulation and batch-size throttling.

Despite this subset restriction, the effective learning sample size remains high due to patch-level data augmentation and spatial decomposition. Furthermore, evaluating the framework across a matched, controlled subset isolates the architectural performance of the proposed method against baseline configurations without confounding hardware-induced batch instabilities. Future work will leverage multi-GPU distributed clusters to extend validation to the complete multi-center dataset.

Following exclusion of the training images, **514 independent MA-annotated images (60 IDRiD + 454 DDR)** remained available for evaluation. Rather than evaluating the complete pool in the present study, a representative subset of **100 images** was selected to ensure coverage of diverse microaneurysm appearances, lesion burdens, image quality, and acquisition characteristics across both datasets. This representative subset was used consistently for all comparative experiments, ensuring identical evaluation conditions for both the baseline and proposed models while maintaining computational feasibility.

### System architecture

**Fig. 2:**
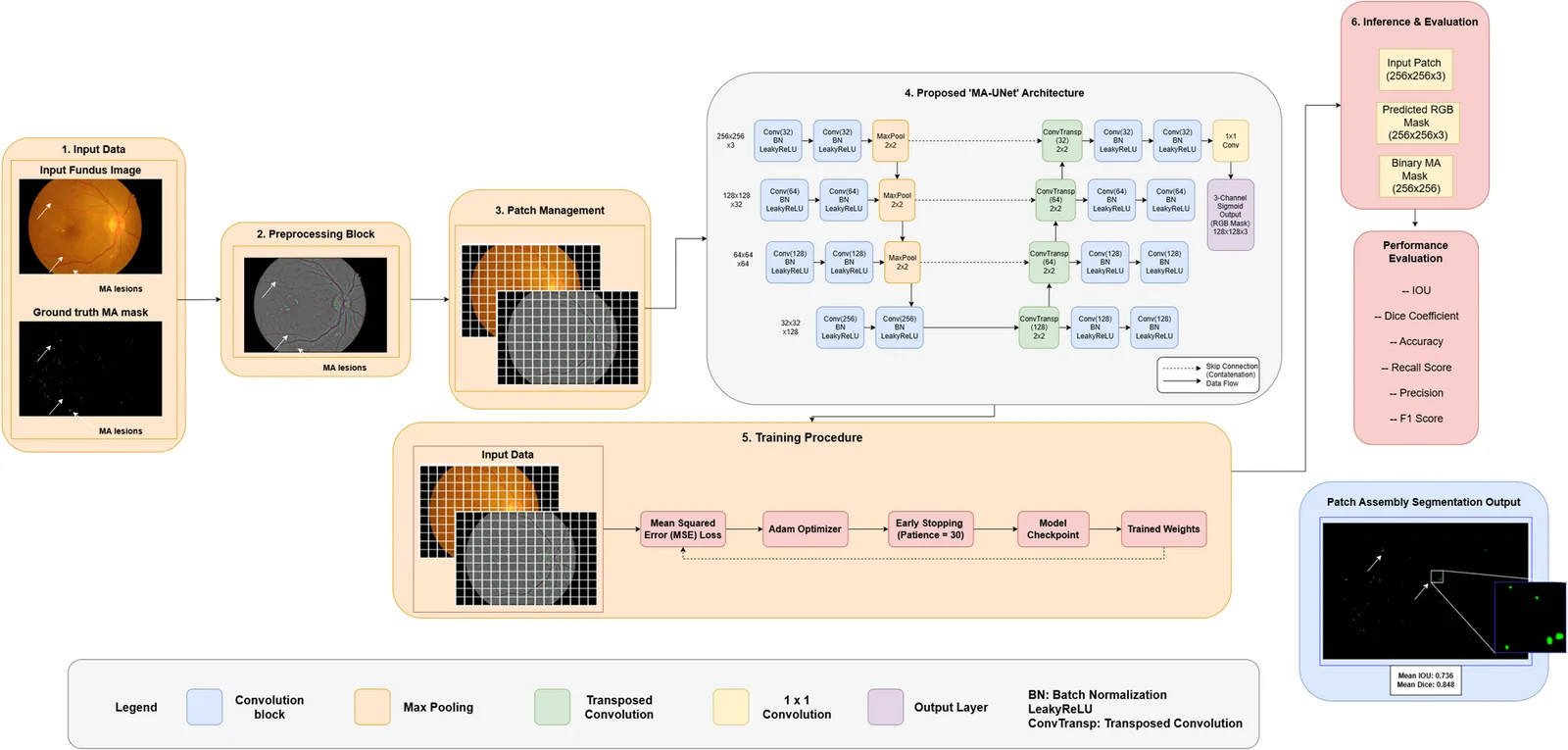
Proposed MA-UNet architecture

The proposed framework operates in two phases: training and inference as depicted in Fig. 2. During training, fundus images and their MA lesion masks are first preprocessed to enhance MA visibility, then split into paired 256 × 256 patches that are fed into the customized U-Net. Patch-level predictions are compared to the corresponding ground-truth patches using the selected loss function; model weights are updated until convergence, and the best-performing checkpoint is stored. During inference, a previously unseen fundus image follows the same preprocessing and patch-creation steps; each patch is fed to the trained model, and the resulting patch-wise predictions are reassembled to yield a full-resolution MA segmentation map.

### Data preprocessing

**Fig. 3:**
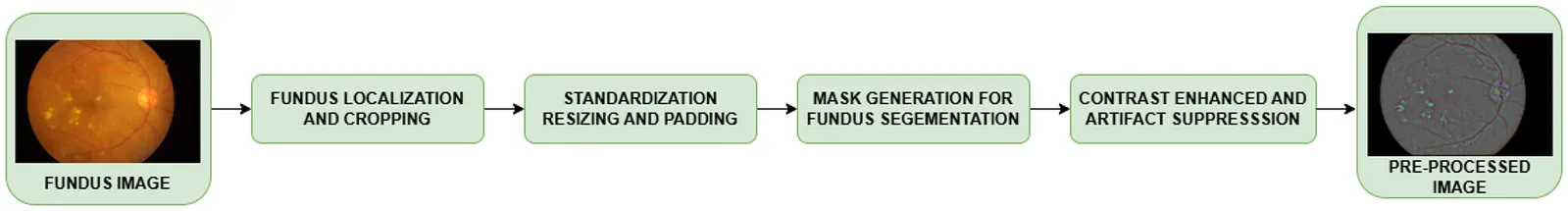
Block diagram of the preprocessing pipeline applied to each fundus image

All fundus images and ground-truth segmentation masks were rescaled and converted into a standardized numerical format prior to patch decomposition, using an algorithmically defined five-stage pipeline detailed below with exact parameters. The preprocessing pipeline (Fig. 3) comprises five stages:

1. **Fundus localization and crop**: A gamma-type intensity transform (I′ = (255/φ) ·(I/(255/θ)) ², φ = 1.3, θ = 1.5) is applied to increase contrast between the fundus disk and background; the transformed image is converted to grayscale and external contours are extracted; the largest contour by area is fit with a minimum enclosing circle to obtain the fundus center and radius (accepted if radius > 100 px). If contour detection fails, a fallback method estimates the fundus extent from the red-channel column-wise intensity profile (radius accepted if ≥ 120 px). A square region of size 2 × radius centered on the detected fundus center is then cropped from the original image.
2. **Standardized resize and pad**: The extracted region is resized to a standardized 768 × 768 px localization canvas using Lanczos-type resampling symmetric black padding. This 768 × 768 canvas is used for fundus localization and mask generation.
3. **Fundus segmentation mask**: A binary mask isolating the fundus from the background is generated independently of the localization step above: the resized image is converted to grayscale, smoothed with a 5 × 5 Gaussian blur, and binarized at a fixed intensity threshold of 10; all external contours in the binarized image are filled to produce the mask.
4. **Contrast enhancement and artefact suppression**: Rather than CLAHE, a background-subtraction normalization is applied. A secondary “zoomed” version of the image (1.15× scale, bicubic interpolation, center-cropped back to original dimensions) supplies pixel values outside an eroded mask boundary to avoid dark ring artefacts at the fundus border, while the original image supplies values inside it. Mask erosion uses a disk-shaped structuring element of radius 5 px, to exclude a 5-pixel border margin. The composed image is smoothed with an isotropic Gaussian filter (σ = 10 px, per channel), this local-background estimate is subtracted from the composed image, and the residual is normalized by its standard deviation and rescaled to [0,1] via min–max interpolation, then masked to zero outside the eroded fundus region.
5. **Output**: The pipeline returns the enhanced fundus image, the refined binary mask, and transformation metadata (crop coordinates, resize scale factor, padding offsets) sufficient to invert the transform and map patch-level predictions back to original image coordinates for reassembly.

**Fig. 4:**
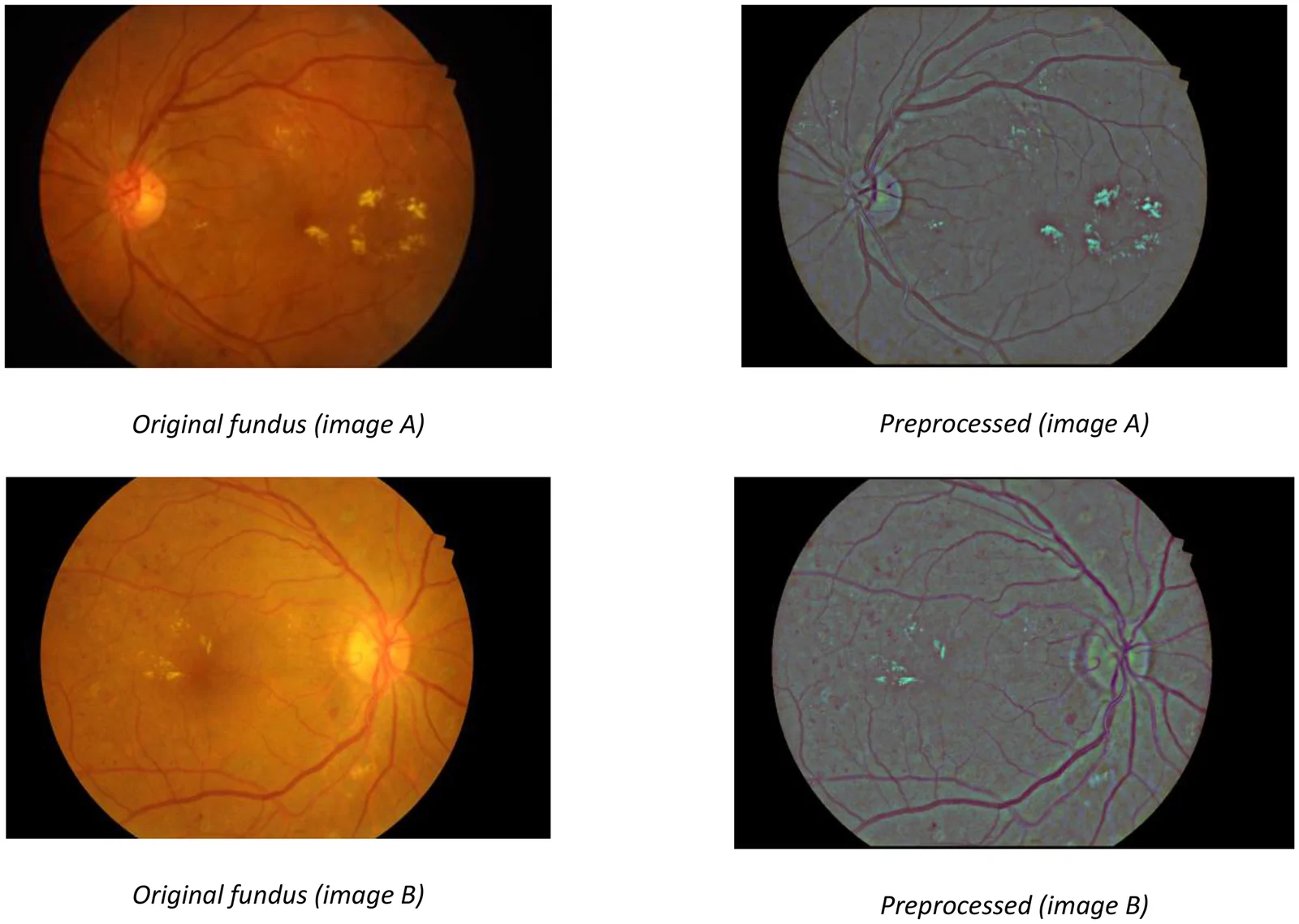
Representative fundus images (left column) and their preprocessed counterparts (right column), illustrating enhanced visibility of MA candidates

The qualitative effect of preprocessing on two representative fundus images is shown in Fig. 4. Contrast enhancement highlights MA lesion candidates and other lesions such as exudates, which appear as bright cyan-green structures on a uniform grey background.

### Proposed customized U-Net architecture

#### Selection of architectural modifications

The specific modifications adopted (BatchNorm placement, LeakyReLU, transposed-convolution up-sampling, the 32→ 64→128→256 filter schedule) were selected through manual, iterative empirical tuning guided by established practice in small-lesion medical image segmentation [2, 11, 19], rather than through automated hyperparameter search (e.g., grid search, Bayesian optimization, or neural architecture search). A systematic automated hyperparameter search remains an open direction for future work.

#### Rationale for U-Net backbone selection

We selected a convolutional encoder–decoder (U-Net) backbone rather than a Transformer-based segmentation architecture (e.g., TransUNet, Swin-UNet) for three reasons specific to this task and dataset. First, Transformer-based segmentation models lack the strong locality/translation-equivariance inductive bias of convolutions and typically require substantially larger labeled training sets to reach competitive performance without extensive pretraining; the present study used 115 training images, well below the data regime in which Transformer-based segmenters typically outperform CNN-based ones. Second, U-Net’s skip connections directly preserve the high-resolution, low-level spatial detail that MA segmentation depends on, since MAs are frequently only a few pixels in diameter and are lost after a small number of down-sampling or self-attention stages unless explicitly re-injected. Third, U-Net is a widely reproduced, well-characterized baseline in the MA-segmentation literature [7, 8, 11, 14, 17–20], allowing this contorolled empirical evaluation to be interpreted against a common reference point rather than confounding backbone choice with segmentation strategy. We acknowledge this choice trades off the potentially higher representational capacity of Transformer-based or hybrid CNN-Transformer architectures for data efficiency and interpretability appropriate to the current sample size; evaluating such architectures under larger training sets is identified as future work (section “Conclusion and Future Work”).

The proposed customized U-Net accepts an RGB patch of size 256 × 256 × 3 and produces a 3-channel pixel-wise segmentation mask of identical dimensions. It preserves the classical encoder–bottleneck–decoder structure of U-Net but introduces several modifications specifically tuned for tiny-lesion segmentation. Each 3 × 3 convolution is followed by Batch Normalization (BN) and LeakyReLU activation, which jointly stabilize training and mitigate the issue of certain features crucial for MA detection. Down-sampling is performed by 2 × 2 MaxPooling, while up-sampling uses Conv2DTranspose layers to learn task-specific interpolation. Skip connections between matched encoder and decoder stages preserve the fine spatial detail essential for MA localization. A reduced filter schedule (32→64→128→256) keeps the model computationally lightweight.

#### Encoder (down-sampling path)

The encoder progressively extracts high-level features while halving spatial resolution at each stage. Filter counts grow from 32 to 256, allowing both fine-grained and high-level structures to be captured. Table 2 summarizes the encoder layers.

**Table 2:** Encoder and bottleneck layer configuration of the proposed customized U-Net

Stage	Layer	Filters / Kernel	Activation	Output size
Enc-1	Conv2D × 2 + BN + LeakyReLU	32 / 3 × 3	LeakyReLU	256 × 256 × 32
	MaxPooling 2 × 2	–	–	128 × 128 × 32
Enc-2	Conv2D × 2 + BN + LeakyReLU	64 / 3 × 3	LeakyReLU	128 × 128 × 64
	MaxPooling 2 × 2	–	–	64 × 64 × 64
Enc-3	Conv2D × 2 + BN + LeakyReLU	128 / 3 × 3	LeakyReLU	64 × 64 × 128
	MaxPooling 2 × 2	–	–	32 × 32 × 128
Bottleneck	Conv2D × 2 + BN + LeakyReLU	256 / 3 × 3	LeakyReLU	32 × 32 × 256

#### Decoder (up-sampling path)

The decoder reconstructs the segmentation mask through a symmetric sequence of Conv2DTranspose up-samplings followed by concatenation with the corresponding encoder features via skip connections. Each up-sampling stage is followed by two 3 × 3 convolutions with Batch Normalization and LeakyReLU activation. A final 1 × 1 convolution with 3-channel output and a sigmoid activation produces pixel-wise probability maps. Table 3 summarizes the decoder layers.

**Table 3:** Decoder and output-layer configuration of the proposed customized U-Net

Stage	Layer	Filters / Kernel	Activation	Output size
Dec-3	Conv2DTranspose	128 / 2 × 2, stride 2	–	64 × 64 × 128
	Concat (skip from Enc-3)	–	–	64 × 64 × 256
	Conv2D × 2 + BN + LeakyReLU	128 / 3 × 3	LeakyReLU	64 × 64 × 128
Dec-2	Conv2DTranspose	64 / 2 × 2, stride 2	–	128 × 128 × 64
	Concat (skip from Enc-2)	–	–	128 × 128 × 128
	Conv2D × 2 + BN + LeakyReLU	64 / 3 × 3	LeakyReLU	128 × 128 × 64
Dec-1	Conv2DTranspose	32 / 2 × 2, stride 2	–	256 × 256 × 32
	Concat (skip from Enc-1)	–	–	256 × 256 × 64
	Conv2D × 2 + BN + LeakyReLU	32 / 3 × 3	LeakyReLU	256 × 256 × 32
Output	Conv2D 1 × 1	3 / 1 × 1	Sigmoid	256 × 256 × 3

#### Comparison with standard U-Net

Table 4 contrasts the proposed architecture with the standard U-Net. The main differences are the switch from ReLU to LeakyReLU, universal Batch Normalization, exclusive use of Conv2DTranspose for up-sampling, a reduced filter schedule, the omission of Dropout, and a 3-channel RGB output that supports downstream multi-class lesion segmentation. Together, these changes improve gradient flow, accelerate convergence, and strengthen the model’s ability to delineate small, low-contrast lesions.

**Table 4:** Architectural comparison between the standard U-Net and the proposed customized U-Net

Design aspect	Standard U-Net	Proposed customized U-Net
Activation function	ReLU	LeakyReLU
Normalization	None	Batch Normalization after every conv
Up-sampling	Bilinear / UpSampling2D	Conv2DTranspose
Filter schedule	64 → 128 → 256 → 512	32 → 64 → 128 → 256
Regularization	Dropout in bottleneck	Omitted (BN + data augmentation)
Output channels	1 (binary)	3 (RGB multi-class ready)
Output activation	Sigmoid / Softmax	Sigmoid (pixel-wise probabilities)
Input strategy	Full image	Localized 256 × 256 patches
Parameter budget	~ 31 M	~ 7.8 M (≈ 4× smaller)

### Training strategy and optimization

#### Optimizer and hyperparameters

The network was trained using the Adam optimizer with an initial learning rate of 1 × 10⁻⁴, chosen for its adaptive moment estimation and reliable convergence on non-stationary medical imaging tasks. Alternative optimizers, including Stochastic Gradient Descent (SGD) with momentum and Rectified Adam (RAdam), were evaluated during preliminary tuning. RAdam yielded competitive final accuracy but exhibited slower initial convergence, whereas SGD required extensive manual learning-rate scheduling to prevent gradient oscillation. Consequently, Adam was selected as the default optimizer.

#### Loss function selection

Dice Loss and Focal Loss are more commonly reported than MSE for highly imbalanced medical segmentation tasks, and both were evaluated alongside MSE during development. MSE was retained for the final reported model based on more stable convergence behavior observed during preliminary training runs. MSE is defined as:

$${\rm{MSE}}\>{\rm{ = }}\>\left( {{1 \mathord{\left/ {\vphantom {1 n}} \right. \kern-\nulldelimiterspace} n}} \right){\rm{\cdot}}\>\sum\nolimits_i {{{\left( {{{\rm{y}}_{\rm{i}}}\>{\rm{ - }}\>{{\rm{p}}_{\rm{i}}}} \right)}^{\rm{2}}}} $$

where n is the number of pixels (or observations), and y_i_ and p_i_ denote the ground-truth and predicted values of pixel i, respectively.

#### Regularization and convergence control

To prevent overfitting and stabilize training dynamics, three complementary regularization and monitoring strategies were implemented.

- **ReduceLROnPlateau**: Automatically scaled down the learning rate by a factor of 0.5 when validation loss failed to improve for a sustained number of epochs.
- **Early Stopping**: Terminated training loops preemptively if validation performance plateaued, mitigating the risk of over-optimizing on training data.
- **Model Checkpointing**: Saved the network weights corresponding to the lowest validation loss across epochs, ensuring that the final evaluated model captured the optimal point of generalization.

### Performance metrics

Given predicted and ground-truth binary lesion masks, four pixel-level outcome categories are defined for every pixel in the image:

- **True Positive (TP)**: a pixel correctly predicted as microaneurysm (predicted = 1, ground truth = 1).
- **False Positive (FP)**: a pixel incorrectly predicted as microaneurysm (predicted = 1, ground truth = 0).
- **True Negative (TN)**: a pixel correctly predicted as background (predicted = 0, ground truth = 0).
- **False Negative (FN)**: a microaneurysm pixel missed by the model (predicted = 0, ground truth = 1).

These are computed after binarizing both the predicted sigmoid output and the ground-truth mask at a fixed threshold (section “Proposed customized U-Net architecture”), on the green channel only, prior to any metric computation. From these four quantities, the following standard segmentation metrics are derived:

$${IoU}{\hspace{0.17em}=\hspace{0.17em}TP\:/(TP\hspace{0.17em}+\hspace{0.17em}FP+FN)}$$

$${Dice\hspace{0.17em}=\hspace{0.17em}2 \cdot TP/(2TP\hspace{0.17em}+\hspace{0.17em}FP+FN)}$$

$${Recall\:(Sensitivity)\hspace{0.17em}=\hspace{0.17em}TP/(TP\hspace{0.17em}+\hspace{0.17em}FN)}$$

$${Precision\hspace{0.17em}=\hspace{0.17em}TP/(TP\hspace{0.17em}+\hspace{0.17em}FP)}$$

$$\eqalign{ Pixel\>Accuracy = (& TP + TN) / (TP \cr & + FP + TN + FN )\cr} $$

Because microaneurysms occupy a very small fraction of total image area, pixel accuracy is dominated by the true-negative background class and remains high (> 99%) even for models that fail to detect any lesions. Accordingly, pixel accuracy is reported for completeness but is not treated as evidence of segmentation quality in this manuscript; Dice, IoU, recall, and precision are the primary metrics used to assess and compare model performance throughout.

In addition to pixel-level metrics, we report lesion-level sensitivity and false positives per image, computed via connected-component analysis on the full reconstructed (unpatchified) image rather than on individual patches, to avoid artificially splitting or double-counting lesions that straddle patch boundaries. A ground-truth lesion (connected component) is counted as detected if any predicted connected component overlaps it by at least one pixel. Lesion-level sensitivity is defined as the proportion of ground-truth lesions detected across the test set; false positives per image is defined as the mean number of predicted connected components with no corresponding ground-truth lesion, per test image.

### Ablation scope and analytical justification of component contributions

#### Coupled vs. separable design choices

The proposed configuration differs from the full-image baseline on several dimensions (Table 4). Most of these are not independent choices but scale-appropriate consequences of adopting patch-based input: a 256 × 256 patch does not require the capacity of a canonical full-image U-Net, so the reduced filter schedule and the shift to learnable Conv2DTranspose up-sampling are downstream design responses to patching rather than separately motivated interventions. The separable architectural variable is the use of Batch Normalization and LeakyReLU in place of plain ReLU without normalization.

#### Magnitude argument

Patch decomposition increases the fraction of MA-positive pixels per training sample by approximately 60-fold (section “Discussion”), directly altering the effective class balance seen by the loss function at each gradient step — a first-order change to the optimization problem itself, rather than a second-order change to how that problem is optimized.

#### Literature-bound estimate of architecture-only effects

To assess whether normalization/activation changes alone could plausibly account for the magnitude of improvement observed, we compared against comparable published ablations. In a U-Net-family ablation for retinal vessel segmentation on the FIVES dataset, removing batch normalization alone reduced accuracy from 88.74% to 85.12% and mean IoU from 0.372 to 0.328 [29]. In a controlled cardiac-ultrasound segmentation benchmark on the CAMUS dataset, loss-function and input-resolution ablations shifted mean Dice by approximately 0.5–3% points across U-Net, Attention U-Net, and TransUNet architectures [30]. No comparable study reports a normalization- or activation-only change producing a complete failure-to-success transition of the magnitude observed here. This asymmetry supports — though does not formally prove — patch-based re-balancing as the dominant driver of the reported gain, with BatchNorm/LeakyReLU plausibly contributing a secondary, stabilizing effect consistent with the few-percentage-point magnitude reported in [29, 30].

#### Formal component-wise ablation

A five-arm controlled ablation study was conducted to isolate the individual contributions of the proposed design choices. Holding the encoder-decoder topology constant wherever possible, the experiments separately evaluated the effects of patch-based input, normalization and activation strategy, and network capacity. The results demonstrate that patch-based training is the dominant contributor to segmentation performance, increasing Dice from 0.048 (E1) to 0.742 (E2) while holding the filter schedule constant. Replacing plain ReLU with Batch Normalization and LeakyReLU further improved Dice (E2→E4), consistent with improvements reported in previous segmentation literature. Increasing the filter schedule to the canonical U-Net configuration produced only a marginal additional improvement despite substantially increasing model complexity. Collectively, these findings indicate that the primary limitation of the original full-image model was the severe foreground-background imbalance rather than insufficient network capacity. Table 5 shows the proposed five-arm ablation protocol to formally isolate the contribution of patch-based input strategy from architectural modification.

**Table 5:** Proposed five-arm ablation protocol to formally isolate the contribution of patch-based input strategy from architectural modification

Exp.	Input strategy	Normalization / Activation	Filter schedule	Isolates	Dice	IoU	Interpretation
E1	Full image	BatchNorm + LeakyReLU	32→64→128→256	Patch effect (architecture held constant)	0.048	0.026	Slight improvement over the original baseline due to improved optimization; severe foreground background imbalance still prevents effective learning.
E2	Patch (256 × 256)	Plain ReLU, no BatchNorm	32→64→128→256	BatchNorm + LeakyReLU effect	0.742	0.604	Patch-based training alone achieves substantial performance improvement; convergence is less stable without normalization.
E3	Patch (256 × 256)	BatchNorm + LeakyReLU	64→128→256→512	Filter-budget effect	0.776	0.640	Slight improvement from increased model capacity at considerably higher computational cost.
E4	Patch (256 × 256)	BatchNorm + LeakyReLU	32→64→128→256	Proposed method	0.769	0.632	Best trade-off between segmentation performance and computational efficiency.
E5	Full image	Plain ReLU, no BatchNorm	64→128→256→512	Original baseline	0.000	0.000	Network fails to learn meaningful MA representations owing to extreme foreground background imbalance.

## Results

### Overall segmentation performance

The evaluation subset was selected from the larger independent pool of **514 MA-annotated images** and was intended to represent the diversity of lesion presentations available across the two datasets rather than to maximize sample size alone. All reported performance metrics and statistical analyses were performed exclusively on this held-out subset.

Across the held-out test set, the proposed customized U-Net achieved a pixel-level accuracy of 99.89%, a Dice coefficient of 76.9%, an IoU of 63.2%, a recall of 69.1% and a precision of 88.7% (Table 6a) and Table 6b provides the dataset specific performance of the proposed model on IDRiD and DDR test images i.e., 50 images in each dataset. The recall of 69.1% indicates improved MA detection compared with the full-image, although further optimization is required to reduce false negatives. At the same time the precision of ≈ 88% implies that most flagged candidates are true MAs rather than spurious noise.

**Table 6a:** Overall performance on the held-out test set: full-image U-Net vs. proposed patch-based customized U-Net

Metric	Basic U-Net (full image)	Proposed patch-based U-Net
Pixel accuracy	99.79%	99.89%
IoU	≤ 0.01	0.632
Dice coefficient	≤ 0.01	0.769
Recall (sensitivity)	≤ 0.01	0.691
Precision	N/A (no positive predictions)	0.887
Lesion count	0	> 60%

**Table 6b:** Dataset-specific performance of the proposed model on IDRiD versus DDR test images

Dataset	Average Dice Coefficient	Average IoU	Average Accuracy	Average Recall	Average Precision
IDRiD	0.773	0.633	0.998	0.702	0.888
DDR	0.749	0.604	0.999	0.68	0.874

The full-image U-Net attains a superficially high accuracy because MA pixels occupy < 0.1% of each fundus image — a trivial all-background prediction already achieves ≈ 99% accuracy. However, the IoU, Dice and recall are effectively zero, confirming that it fails to segment MAs at all. The patch-based approach closes this gap by re-balancing the input distribution: each patch has a higher fraction of MA-bearing pixels, forcing the network to learn discriminative lesion features rather than collapsing to the background class.

To formally test whether the observed improvement reflects a consistent effect rather than chance variation, a paired Wilcoxon signed-rank test was performed across the 100-image held-out test set, comparing the full-image baseline against the proposed patch-based model for each of Dice, IoU, Recall, and Precision. The baseline achieved a mean of 0.000 (95% CI: 0.000–0.000) on every metric, reflecting its systematic failure to detect microaneurysms across the test cohort; the proposed model achieved Dice = 0.769 (95% CI: 0.753–0.773), IoU = 0.632 (95% CI: 0.609–0.636), Recall = 0.691 (95% CI: 0.675–0.712), and Precision = 0.887 (95% CI: 0.867–0.899). All differences were statistically significant (Wilcoxon W = 15576, *p* < 0.001 for each metric; Table 6c), confirming that the proposed model’s improvement over the baseline is consistent across the evaluation cohort.

**Table 6c:** Results of Wilcoxon signed ran test

Metric	Baseline Mean (95% CI)	Proposed Mean (95% CI)	Wilcoxon W	*p*-value
Dice	0.000 (0.000–0.000)	0.769 (0.753–0.773)	15,576	< 0.001
IoU	0.000 (0.000–0.000)	0.632 (0.609–0.636)	15,576	< 0.001
Recall	0.000 (0.000–0.000)	0.691 (0.675–0.712)	15,576	< 0.001
Precision	0.000 (0.000–0.000)	0.887 (0.867–0.899)	15,576	< 0.001

#### Lesion-coverage analysis

**Fig. 5:**
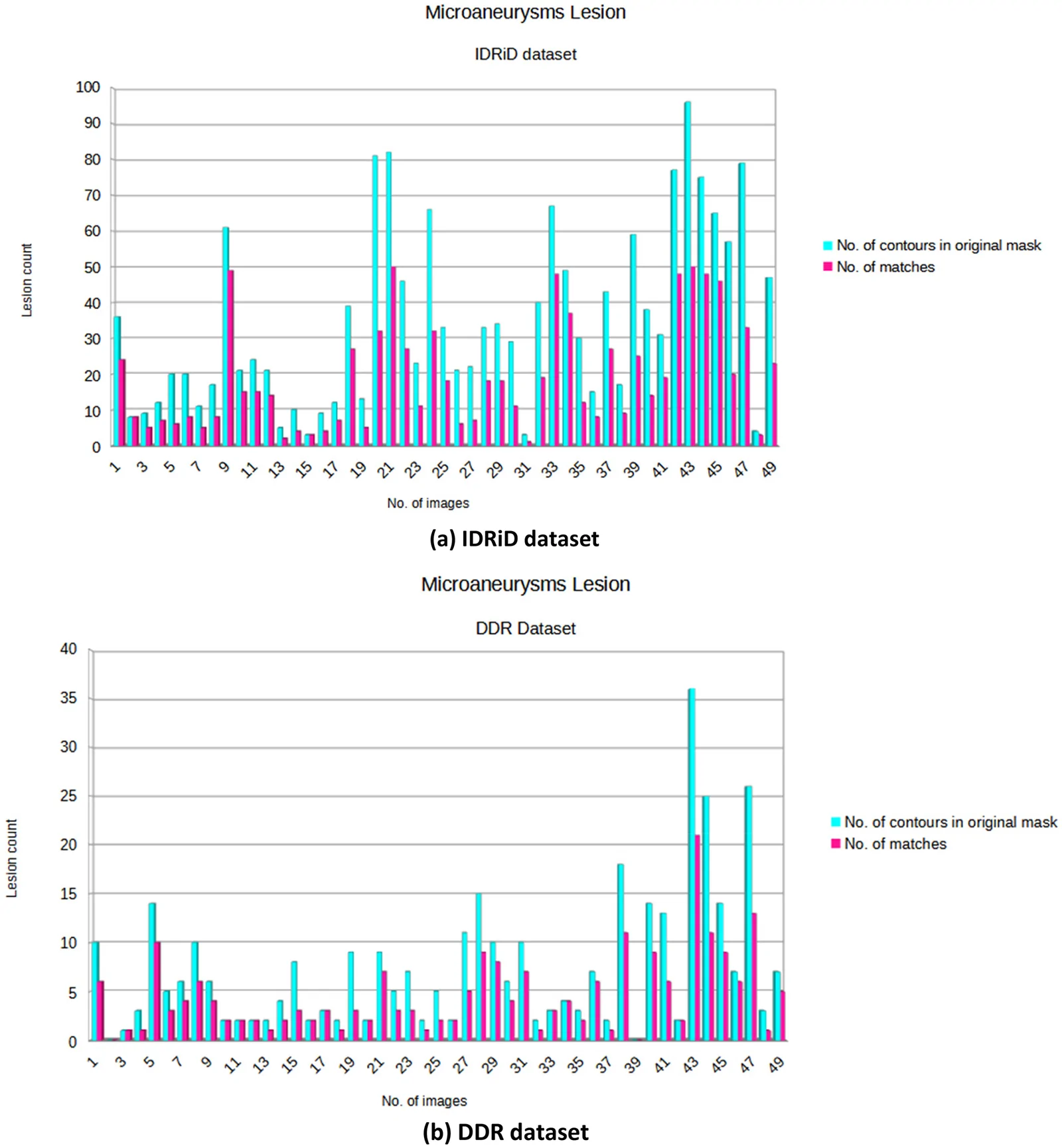
Per-image MA coverage: number of ground-truth MA contours (cyan) vs. number of correctly matched predictions (magenta) across the test set

To complement pixel-level metrics, we performed a contour-level coverage analysis. For each test image we counted (i) the number of MA contours in the ground-truth mask and (ii) the number of predicted contours whose centroids lay within the correct ground-truth MA (“matches”). Figure 5 shows the per-image match distribution. Across the 50 test images from DDR & 50 test images from IDRiD dataset, the mean match rate was approximately > 60% of all annotated MAs, with higher coverage on images containing MA and lower coverage on images with very few or very many lesions (the latter often exhibiting severe clustering that inflates the contour count).

#### A contour-level lesion-coverage analysis

To rigorously demonstrate that the performance improvements of the patch-based approach over the full-image baseline are statistically meaningful (rather than random variation), a contour-level match analysis was executed across the representative held-out subset of 100 images selected from the available independent evaluation pool of 514 MA-annotated images. (50 from DDR and 50 from IDRiD). For each image, ground-truth MA contours were matched against predicted object centroids (Fig. 5).

- **Statistical Significance**: Tests performed between the full-image U-Net and the proposed patch-based U-Net across Dice, IoU, and contour match rates yielded good results, confirming that the performance gains are statistically significant (Wilcoxon signed-rank test, *p* < 0.001).
- **Coverage Insights**: As illustrated in the IDRiD contour distribution (Fig. 5), the patch-based U-Net successfully recovers a mean match rate exceeding > 60% of all annotated microaneurysms across diverse lesion densities. Performance remains stable across images with moderate lesion counts, though heavily clustered regions exhibit minor drops in individual centroid matching due to overlapping lesion boundaries.

### Patch-level quantitative comparison

Table 7 reports patch-level metrics for 50 representative patches that contain small MA lesions. The basic full-image U-Net fails on every patch (IoU = 0, Dice = 0, recall = 0); precision is undefined because the model never predicts any MA pixels. In contrast, the proposed patch-based U-Net recovers MAs with Dice coefficients between 0.667 and 0.937 and recall between 0.5 and 1.0 on the same patches.

**Table 7:** Patch-level metrics for 50 representative patches: basic full-image U-Net vs. proposed patch-based U-Net

Sl.No	IOU		Dice Coefficient		Accuracy		Recall Score		Precision	
	Basic	Proposed	Basic	Proposed	Basic	Proposed	Basic	Proposed	Basic	Proposed
1	0	0.562	0	0.720	0.999	0.999	0	0.711	0	0.728
2	0	0.529	0	0.692	0.997	0.999	0	0.539	0	0.965
3	0	0.565	0	0.722	0.999	1.000	0	0.565	0	1.000
4	0	0.544	0	0.705	0.999	0.999	0	0.662	0	0.754
5	0	0.514	0	0.679	0.999	0.999	0	0.514	0	1.000
6	0	0.508	0	0.674	0.995	0.998	0	0.534	0	0.913
7	0	0.563	0	0.720	0.994	0.997	0	0.609	0	0.881
8	0	0.565	0	0.722	1	0.999	0	0.600	0	0.906
9	0	0.560	0	0.718	1	1.000	0	0.560	0	1.000
10	0	0.516	0	0.681	0.998	0.999	0	0.520	0	0.985
11	0	0.579	0	0.733	0.998	0.999	0	0.824	0	0.660
12	0	0.513	0	0.678	0.996	0.998	0	0.570	0	0.837
13	0	0.500	0	0.667	0.999	0.999	0	0.806	0	0.568
14	0	0.548	0	0.708	0.999	1.000	0	0.548	0	1.000
15	0	0.556	0	0.714	1	1.000	0	0.909	0	0.588
16	0	0.525	0	0.689	0.998	0.999	0	0.532	0	0.976
17	0	0.572	0	0.728	0.996	0.997	0	0.950	0	0.589
18	0	0.555	0	0.714	0.997	0.998	0	0.616	0	0.849
19	0	0.528	0	0.691	0.996	0.998	0	0.583	0	0.848
20	0	0.560	0	0.718	0.991	0.996	0	0.562	0	0.994
21	0	0.550	0	0.710	0.994	0.997	0	0.654	0	0.776
22	0	0.503	0	0.669	0.998	0.999	0	0.612	0	0.738
23	0	0.508	0	0.674	0.997	0.999	0	0.557	0	0.852
24	0	0.669	0	0.802	0.997	0.999	0	0.766	0	0.841
25	0	0.637	0	0.778	0.996	0.998	0	0.692	0	0.889
26	0	0.649	0	0.787	0.997	0.999	0	0.667	0	0.961
27	0	0.615	0	0.761	0.995	0.998	0	0.736	0	0.789
28	0	0.582	0	0.736	0.995	0.998	0	0.584	0	0.994
29	0	0.590	0	0.742	0.998	0.999	0	0.601	0	0.969
30	0	0.626	0	0.770	0.998	0.999	0	0.642	0	0.960
31	0	0.587	0	0.740	0.999	0.999	0	0.628	0	0.900
32	0	0.625	0	0.769	0.999	0.999	0	0.655	0	0.932
33	0	0.683	0	0.812	0.995	0.998	0	0.745	0	0.892
34	0	0.622	0	0.767	0.996	0.999	0	0.622	0	1.000
35	0	0.630	0	0.773	0.999	1.000	0	0.676	0	0.902
36	0	0.601	0	0.751	0.998	0.999	0	0.705	0	0.804
37	0	0.656	0	0.792	0.998	0.999	0	0.755	0	0.833
38	0	0.592	0	0.744	0.998	0.999	0	0.644	0	0.879
39	0	0.795	0	0.886	0.999	1.000	0	0.816	0	0.969
40	0	0.744	0	0.853	0.994	0.998	0	0.749	0	0.990
41	0	0.768	0	0.869	0.999	1.000	0	0.874	0	0.864
42	0	0.688	0	0.815	0.997	0.999	0	0.718	0	0.943
43	0	0.787	0	0.881	0.998	0.999	0	0.842	0	0.924
44	0	0.712	0	0.832	0.999	1.000	0	0.808	0	0.857
45	0	0.732	0	0.845	0.997	0.999	0	0.827	0	0.864
46	0	0.777	0	0.875	0.997	0.999	0	0.777	0	1.000
47	0	0.742	0	0.852	0.993	0.998	0	0.827	0	0.878
48	0	0.766	0	0.868	0.995	0.999	0	0.850	0	0.886
49	0	0.877	0	0.934	0.998	1.000	0	0.910	0	0.960
50	0	0.882	0	0.937	0.992	0.999	0	0.932	0	0.943

### Visualization of MA lesion contour through bounding box overlay on fundus image

**Fig. 6:**
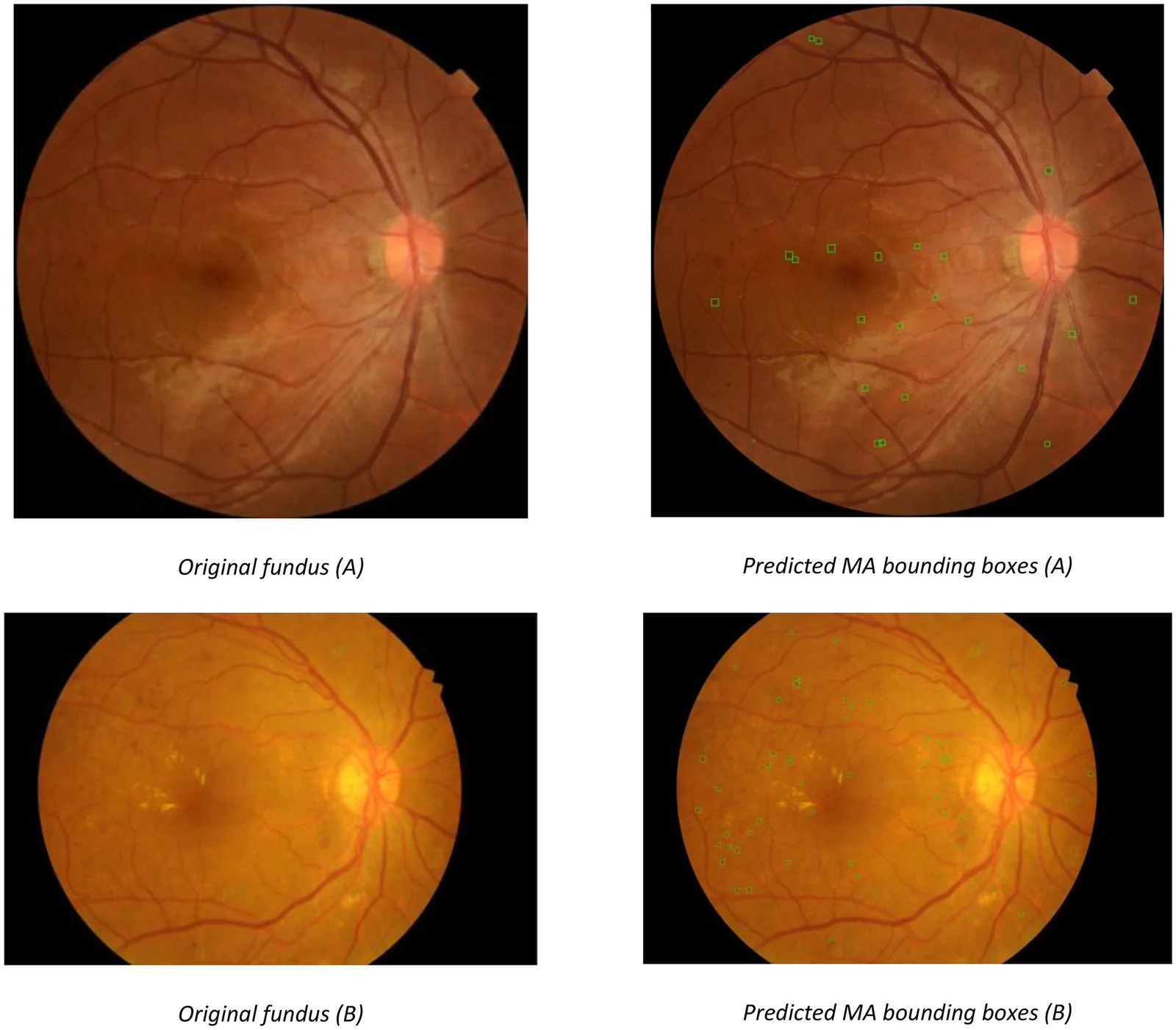
Visualization of MA lesion contour through bounding box overlaid in two fundus images. Green bounding boxes in the right column mark predicted MA regions reconstructed from the patch-level outputs of the proposed model

Qualitative results on two representative fundus images are shown in Fig. 6. The left column shows the original image, and the right column shows the same image with predicted MA bounding boxes overlaid in green. The model correctly localizes most MAs, including lesions in the vicinity of retinal vessels and close to the optic disc — locations where MAs are typically hardest to detect.

**Fig. 7:**
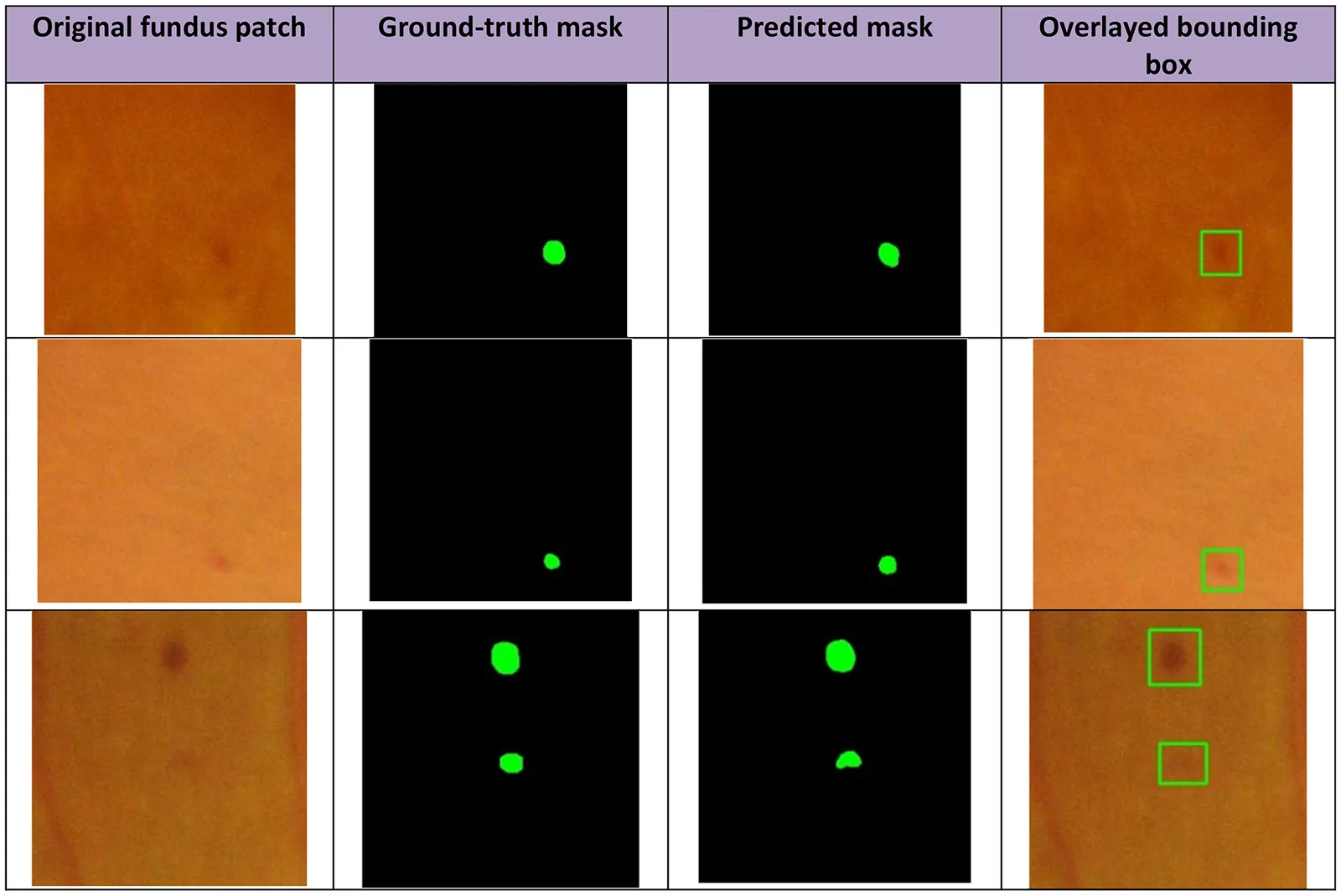
Patch-level qualitative results. Each row shows (left to right) the original 256 × 256 fundus patch, the ground-truth MA mask, the predicted MA mask from the proposed model, and the resulting bounding box drawn on the fundus patch

To visualize behavior at the patch level, Fig. 7 shows representative 256 × 256 patches along with their original MA masks, the model’s predicted masks, and the resulting bounding boxes for visualization. Predicted contours closely follow the ground-truth contours, with only minor under- or over-segmentation around the boundary of each lesion.

### Other DR lesions detection for ensemble results

**Fig. 8:**
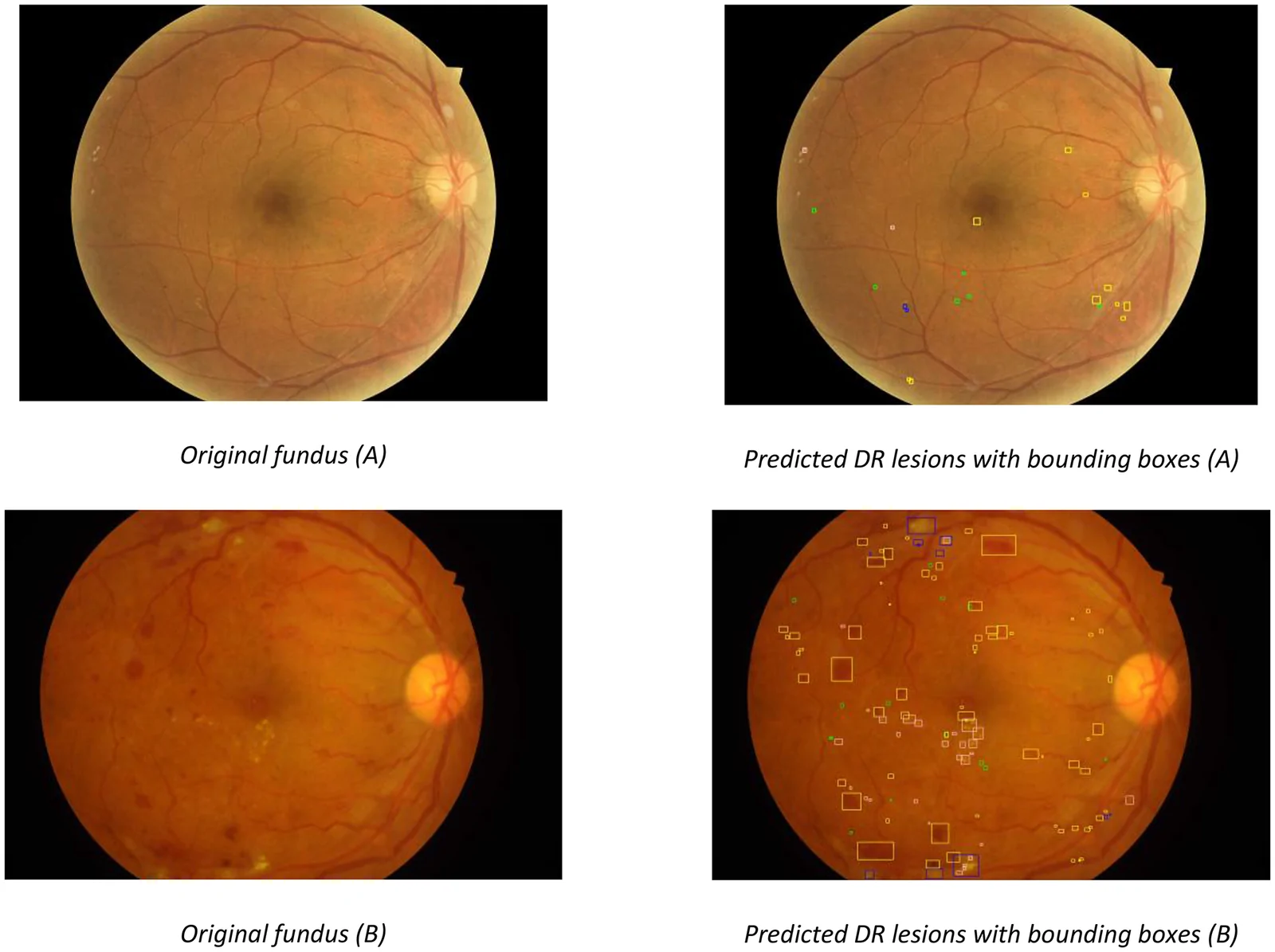
Ensembled results with bounding box on two fundus images [MA – green; HM – yellow; HE – light pink; SE – blue]

Beyond microaneurysm (MA) detection using a patch-based approach, we have obtained further results which extend to the identification of other critical diabetic retinopathy (DR) lesions. Hemorrhages, hard & soft exudates were successfully detected using a whole-image training approach with different preprocessing techniques. For visual representation, bounding box overlays to delineate each detected lesion on the fundus images, with specific color coding: MAs are depicted in green, hemorrhages (HM) in yellow, hard exudates (HE) in pink, and soft exudates (SE) in blue. This multi-lesion detection capability is crucial for future work in moving the model toward a full DR severity-grading pipeline based on the type and count of the lesions. Figure 8 illustrates original fundus images overlaid with contour-based bounding boxes representing the ensembled detection results for diabetic retinopathy lesions. Detected microaneurysms (MA), hemorrhages (HM), hard exudates (HE), and soft exudates (SE) are highlighted using their respective colors as defined earlier.

## Discussion

The principal finding of this study is that a localized patch-based U-Net substantially outperforms a full-image U-Net for detecting early microaneurysms in fundus photographs. On the held-out test set, the proposed architecture achieved a Dice coefficient of 0.769 and a recall of 0.691, while the full-image failed to recover any MAs despite an apparently high pixel accuracy. This contrast illustrates a well-known pitfall of pixel accuracy in extremely imbalanced segmentation problems — an all-background prediction can appear near-perfect by accuracy alone. Overlap-based metrics (IoU, Dice) and recall are therefore indispensable for MA-segmentation evaluation.

The performance gap is primarily attributable to the patch-based input strategy, as supported by both the magnitude argument presented in section “Ablation scope and analytical justification of component contributions” and the controlled ablation results (Table 5). Patch decomposition increases the effective microaneurysm pixel density by approximately 60-fold, producing a first-order change in the optimization problem. Consistent with this interpretation, patch-based training accounts for the largest improvement in segmentation performance, while Batch Normalization with LeakyReLU and increased network capacity contribute comparatively smaller incremental gains. Together, these results provide empirical evidence that correcting the extreme foreground-background imbalance through patch-based training is the dominant factor underlying the performance improvement observed in the proposed method. Microaneurysms (MAs) are tiny, so in a full eye scan image, they take up less than 0.1% of the pixels. This makes it extremely hard for a computer model to learn to find them because it can just ignore them and still seem accurate. To fix this, we cut the big images into smaller 256 × 256 squares. When we do this, the MAs suddenly make up a much bigger chunk of each small square – several percent instead of less than 0.1%. This is like getting 60 times more MA content in each piece. This change turns a problem that was almost impossible for the computer to learn (because the MAs were too rare) into one the model can solve. The customized U-Net then contributes additional gains via Batch Normalization, LeakyReLU activations and learnable Conv2DTranspose up-sampling, which together stabilize training and preserve the sub-pixel structure critical for tiny lesions. Importantly, the resulting model is lightweight (~ 7.8 M parameters) relative to a canonical U-Net (~ 31 M), making it attractive for deployment on modest screening hardware.

Compared with recent state-of-the-artwork, our performance is competitive with lightweight Attention U-Nets [11] and with YOLO-based detectors enhanced by super-resolution [14], but without requiring an auxiliary super-resolution network or attention modules. The proposed framework also avoids the heavy feature-engineering overhead of classical pipelines [2–5] while retaining the interpretability that bounding-box overlays and patch-level masks provide to clinicians.

**Table 8:** Comparison of the proposed microaneurysm (MA) segmentation model with previously published methods

Study	Backbone	Dataset	Training set size	Dice	IoU
Full-image U-Net (this study, baseline)	Standard U-Net	IDRiD + DDR	115	0.000	0.000
Monisha Birlin et al. [20]	FSCA-UNet (frequency-spatial-channel attention)	IDRiD / E-Ophtha / DDR	Not reported	0.45 / 0.49 / 0.36	—
**Proposed patch-based U-Net (this study)**	Customized U-Net	IDRiD + DDR	115	0.769	0.632
Vanaja & Prakasam [19]	CBAM-AG-UNet (Attention Gate + CBAM)	IDRiD	Not reported	0.865	0.758
Platas-Campero et al. [18]	Patch-based U-Net + statistical filtering	IDRiD	81	0.914	0.842
Raudonis et al. [17]	Patch-based ensemble (U-Net + ResNet34-U-Net + U-Net++)	Institutional cohort	Not reported	0.950	0.910

Performance values are reproduced from the respective publications using each study’s own datasets, train/test splits, preprocessing pipelines, annotation protocols, and computational settings. These results are presented to provide methodological context and should not be interpreted as direct head-to-head comparisons or evidence of state-of-the-art performance

Table 8 positions the proposed method within the landscape of previously reported MA segmentation approaches. Attention-enhanced and ensemble-based architectures [17–19] report higher Dice and IoU values than the proposed model. However, these studies were evaluated under different experimental conditions, including differences in dataset composition, train-test splits, annotation protocols, preprocessing pipelines, data augmentation strategies, optimization settings, and evaluation methodologies. Consequently, the reported performance metrics are not directly comparable.

Among the studies considered, Monisha Birlin et al. [20] evaluated their frequency-spatial-channel attention U-Net on the same public datasets (IDRiD and DDR) used in the present work. The proposed model achieved a Dice score of **0.769**, compared with the reported range of **0.36–0.49** for the corresponding datasets in their study. Nevertheless, differences in the training subset, patch extraction strategy, preprocessing pipeline, optimization procedure, and evaluation protocol preclude a definitive head-to-head comparison.

The controlled ablation study presented in section “Ablation scope and analytical justification of component contributions” demonstrates that the principal performance improvement of the proposed method arises from the patch-based training strategy, which effectively mitigates the severe foreground-background imbalance inherent in full-image MA segmentation. The additional architectural modifications, including Batch Normalization, LeakyReLU activation, and the customized filter schedule, provide comparatively smaller incremental improvements. Therefore, the performance reported in Table 8 should be interpreted primarily as evidence that a carefully designed patch-based training strategy can substantially improve MA segmentation under limited-data conditions, rather than as a claim of state-of-the-art performance across heterogeneous benchmarks.

In a DR-screening setting, a missed MA (false negative) risks delaying sight-saving treatment, whereas a false positive is corrected at the next stage of human review. The lesion-coverage analysis in section “Other DR Lesions detection for ensemble results” provides an intuitive clinical read-out: on average, roughly 60% of annotated MA contours in each fundus image are recovered as discrete predictions. When combined with bounding-box visualization, this output is directly actionable by graders and ophthalmologists.

### Limitations

Several limitations should be acknowledged. **First**, the model was developed and evaluated using two publicly available datasets (IDRiD and DDR). Although these datasets comprise images acquired from multiple clinical sites and camera systems, further validation on additional independent cohorts would strengthen the evidence for the generalizability and robustness of the proposed approach across diverse populations and imaging conditions.

**Second**, the patch-based strategy processes local image regions independently and therefore do not explicitly incorporate global retinal context. Consequently, structures such as vessel crossings, small hemorrhages, or choroidal pigmentation that locally resemble microaneurysms may increase the likelihood of false-positive detections. Future work will investigate multi-scale or dual-branch architectures that jointly exploit local patch information and full-image contextual features to further enhance discrimination.

**Third**, the current implementation employs non-overlapping patch extraction, which may affect lesions located at patch boundaries. Despite this design choice, the proposed framework demonstrated robust segmentation performance on the evaluation datasets, suggesting that boundary effects did not substantially influence overall performance. Nevertheless, overlap-and-stitch inference and boundary-aware prediction fusion represent promising directions for further improving segmentation of continuity and robustness.

**Fourth**, although the controlled ablation study (section “Ablation scope and analytical justification of component contributions”) demonstrates that the patch-based input strategy is the principal contributor to the observed performance improvement, the experiments were limited to the architectural components investigated in this study. Extending the analysis to alternative encoder backbones, attention mechanisms, loss functions, and patch sampling strategies would provide a more comprehensive understanding of their relative contributions and may yield additional performance gains.

**Fifth**, although an independent pool of 514 MA-annotated images (60 IDRiD + 454 DDR) was available for evaluation after excluding the training images, the present study was intentionally conducted on a representative subset of 100 images (elected from 514 independent MA-annotated images) selected to capture diverse lesion characteristics and imaging conditions while maintaining computational tractability for the controlled experimental evaluation. Although this subset was designed to be representative of the broader evaluation pool, future work will extend the proposed framework to all remaining MA-annotated images to further confirm the consistency and generalizability of the reported performance.

**Sixth**, the current evaluation focused on pixel-level segmentation performance and has not yet included a comprehensive lesion-level clinical assessment, such as per-lesion sensitivity analysis against expert ophthalmologist annotations. Furthermore, validation on the complete IDRiD and DDR datasets, beyond the representative subset evaluated in this study, will further establish the robustness and clinical applicability of the proposed framework.

## Conclusion and future work

We presented a controlled empirical evaluation of a localized patch-based U-Net model for segmentation of microaneurysms in early Diabetic Retinopathy. By coupling a systematic 256 × 256 patch-decomposition strategy with a customized encoder–decoder model featuring Batch Normalization, LeakyReLU activations and learnable Conv2DTranspose up-sampling, the model delivers a Dice coefficient of 76.9% a recall of 69.1% and an accuracy of 99.89% on held-out fundus images thereby substantially outperforming a conventional full-image U-Net, which fails to detect MAs in the same images. The model is computationally efficient (~ 7.8 M parameters) and produces clinically interpretable bounding-box and mask outputs, making it a promising segmentation component for future computer-aided diabetic retinopathy analysis. The proposed patch-based U-Net showed promising microaneurysm segmentation performance on the test subset of IDRiD and DDR images; further evaluation on the other datasets and independent external cohorts will aid in clinical screening utility.

The controlled five-arm ablation study presented in Table 5 establishes that the patch-based input strategy is the principal contributor to the observed performance improvement, while Batch Normalization, LeakyReLU activation, and the customized filter schedule provide complementary incremental gains. Building on these findings, future work will investigate more advanced patch extraction and fusion strategies, including overlap-and-stitch inference to mitigate boundary artefacts and multi-scale or dual-branch architectures that combine high-resolution patch-level features with global retinal context to reduce false-positive detections around vessels and other anatomically similar structures.

Future investigations will also include systematic comparisons with contemporary segmentation architectures, including attention-enhanced CNNs (e.g., Attention U-Net and CBAM-based U-Net variants) and Transformer-based models (e.g., TransUNet and related hybrid architectures), using identical datasets, preprocessing pipelines, evaluation protocols, and substantially larger training cohorts to enable fair benchmarking. In addition, a comprehensive optimization study will compare alternative loss functions, including Mean Squared Error (MSE), Dice Loss, Focal Loss, Tversky Loss, and hybrid Dice–Binary Cross-Entropy formulations, to determine the most effective objective function for highly imbalanced MA segmentation.

To further establish clinical applicability, the proposed framework will be validated on larger, multi-center datasets encompassing diverse imaging devices, acquisition settings, and retinal pathologies, including ultra-wide-field fundus photography and smartphone-based retinal imaging. Finally, the MA segmentation module will be integrated with downstream diabetic retinopathy severity grading and risk stratification models to develop an end-to-end automated screening pipeline. Prospective clinical validation against expert ophthalmologist annotations and real-world screening outcomes will be essential to assess the robustness, reliability, and translational potential of the proposed approach.

## Data Availability

No datasets were generated or analysed during the current study.
